# The role of hinged external fixation in the treatment of knee dislocation, subluxation and fracture‐dislocation: A systematic review of indications

**DOI:** 10.1002/jeo2.70275

**Published:** 2025-05-19

**Authors:** Ekrem M. Ayhan, Sarah Levitt, Geoffrey D. Abrams, James P. Stannard, Michael J. Medvecky

**Affiliations:** ^1^ Department of Orthopaedics and Rehabilitation Yale School of Medicine New Haven Connecticut USA; ^2^ Frank H. Netter MD School of Medicine Quinnipiac University North Haven Connecticut USA; ^3^ Department of Orthopedic Surgery Stanford University Stanford California USA; ^4^ Department of Orthopaedic Surgery University of Missouri Columbia Missouri USA; ^5^ Thompson Laboratory for Regenerative Orthopaedics University of Missouri Columbia Missouri USA

**Keywords:** external fixation, external fixator, hinged external fixation, hinged external fixator, indications, knee dislocation, knee fracture‐dislocation, knee subluxation, multiple ligament knee injury

## Abstract

**Purpose:**

While rigid knee‐spanning external fixation is more commonly utilized in the treatment of complex knee injuries compared to hinged external fixation (HEF), HEF has the added benefit of providing stability while also permitting early controlled range of motion. However, there is limited existing literature on the indications for HEF of the knee. The aim of this study was to review the clinical indications for HEF in the management of acute and chronic knee dislocations (KD), tibiofemoral subluxations and knee fracture‐dislocations.

**Methods:**

Five databases, including PubMed, CINAHL, Cochrane, Scopus and SPORTDiscus, were systematically searched. Included studies were those that involved comparative or non‐comparative evaluation of patients with an HEF applied for an acute or chronic KD, tibiofemoral subluxation or knee fracture‐dislocation.

**Results:**

Fourteen studies ranging from 1998 to 2023 met inclusion criteria, with a total of 184 knees treated with HEF for an acute or chronic KD, tibiofemoral subluxation, or knee fracture‐dislocation. The most common primary indication for HEF was acute or chronic KD. The most common secondary indications included combined osseous and ligamentous deficiency, associated vascular or soft‐tissue injury, status post extensive capsular release for arthrofibrosis, and associated extensor mechanism disruption.

**Conclusion:**

While uncommon, HEF is a valuable option in the treatment of complex knee injuries where both stability and controlled mobilization are essential. Due to limitations in the available evidence, further high‐quality research is needed to establish guidelines for the utilization of HEF about the knee.

**Level of Evidence:**

Level IV.

**Abbreviations**
ACL: anterior cruciate ligament
HEF: hinged external fixation
KD: knee dislocation
KSEF: knee‐spanning external fixation
MCL: medial collateral ligament
MINORS: Methodological Index for Nonrandomized Studies
MLKI: multi‐ligamentous knee injury
PCL: posterior cruciate ligament
PLC: posterolateral corner
PMC: posteromedial corner
PRISMA: Preferred Reporting Items for Systematic Reviews and Meta‐Analyses
ROM: range of motion

## INTRODUCTION

Knee dislocations (KD), tibiofemoral subluxations and knee fracture‐dislocations present significant challenges in orthopaedic trauma management due to the dichotomous need for joint stability and mobility. KDs, which are classically defined as the complete separation of normal joint contact between the tibial and femoral articular surfaces, are rare but devastating injuries of the knee joint accounting for 0.03%–0.18% of trauma cases globally [5, 34, 47]. However, severe ligamentous injuries can also occur without a true KD, as seen in most multi‐ligamentous knee injuries (MLKI) [29]. MLKIs, while previously thought of as synonymous with KDs, are distinct injuries involving disruption of at least two of the four major knee ligaments without evidence of a knee joint dislocation [37]. Both KDs and MLKIs are potentially limb‐threatening injuries and are associated with severe complications, including persistent pain, instability and arthrofibrosis [7, 33, 35]. While there appears to be a distinct vascular injury risk associated with documented KDs versus non‐dislocated MLKIs, a high index of suspicion of vascular injury needs to be maintained for both injuries [34]. Knee fracture‐dislocations, classified as KD‐V by the Schenck KD classification system, are an even rarer subset of injuries characterized by total disruption of the tibiofemoral joint with an associated periarticular fracture that increases instability and neurovascular risk [13, 19, 31, 34, 46]. Recent expert consensus has clarified which specific fracture patterns constitute KD‐V injuries [34]. Combined osseous and ligamentous deficiency in the setting of knee fracture‐dislocation makes these injuries difficult to treat secondary to relative time urgency and potentially magnified joint instability [13]. Furthermore, these patients often present in the setting of polytrauma, which may divert care teams toward damage control and away from the fracture‐dislocation [13].

**Figure 1:**
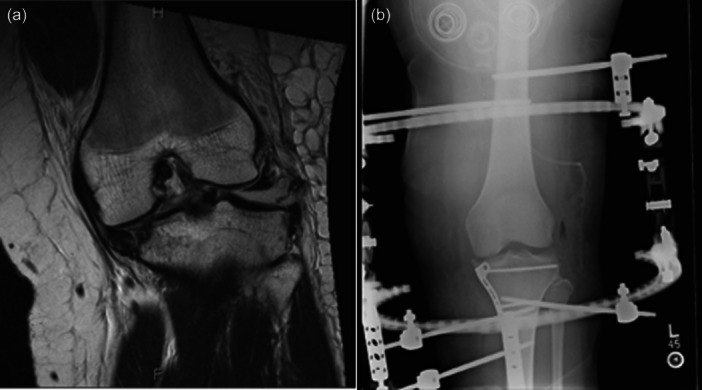
(a) Coronal proton density‐weighted MRI image demonstrating lateral coronal tibiofemoral subluxation, lateral compartment distraction and peripheral rim medial tibial plateau fracture, not shown is the associated posterolateral corner disruption with avulsion of the lateral collateral ligament, biceps femoris and patellofemoral ligament from the fibular head. (b) Post reduction X‐ray with internal fixation of tibial plateau fracture and application of a Compass Hinge External Fixator. MRI, magnetic resonance imaging.

Initial and/or definitive management of these injuries may need to include external fixation, which has the potential risk of arthrofibrosis, stiffness, and pain due to prolonged immobilization and altered joint biomechanics [15, 26, 32, 58]. External fixation may be applied in either a uniplanar or multiplanar configuration, depending upon the needs for stabilization. Hinged external fixators (HEF), originally used in elbow dislocation, offer the advantage of multiplanar stability while also permitting early controlled range of motion (ROM), which may be more advantageous than rigid external fixation in select clinical scenarios [6]. This may allow for earlier joint mobilization, improved multiplanar tibiofemoral support, decreased risk of arthrofibrosis, and more immediate and progressive rehabilitation without the added risk of osseous or ligamentous failure [54]. These devices have been described in the setting of acute KD, chronic or neglected KD, fracture‐dislocation, tibiofemoral subluxation and complex tibial plateau fracture [2, 3, 4, 18, 20, 22, 23, 27, 30, 42, 43, 45, 48, 51, 53, 54, 55, 56, 58]. They are designed to span the knee joint using one or two rigid rings or links, which are fixed to the femur and tibia through direct pin fixation and connected by a hinge [17]. The hinge must have an axis of rotation coincident with the natural flexion‐extension axis of the knee to prevent aberrant forces from being placed onto the ligaments and articular surfaces [22, 23, 52]. Newer designs of HEF devices use the transepicondylar axis of the knee as a reference and aim to reproduce the four‐bar‐linkage model of cruciate ligaments to more effectively imitate natural knee kinematics [58]. Several HEF devices are currently available commercially, including both monolateral and bilateral assemblies, which have performed similarly in biomechanical tests of tibial translation, anterior and posterior drawer tests and Lachman tests [14, 52]. An MRI example of a Compass HEF being used in the setting of a knee fracture‐dislocation can be seen below in Figure 1.

While the data remains largely limited to Levels IV and V studies, several uses for HEF have been described in the treatment of complex knee injuries. For example, protection of articular or ligamentous repairs using HEF during aggressive physical therapy has been shown to improve functional outcomes and potentially prevent arthrofibrosis [3, 28, 30, 48, 55, 58]. This finding is supported by kinematic studies of HEF devices, which are capable of rigid fixation in all planes except sagittal [14, 52]. HEF devices have also been described for the purpose of allowing periarticular soft tissue healing before definitive reconstruction while allowing controlled joint mobilization [20, 30, 54, 56]. This may be particularly important for polytrauma patients during damage control treatment, where associated injuries may need to be stabilized prior to definitive treatment of structures around the knee [18]. There is also evidence that HEF devices are useful in maintaining coronal and axial alignment following reduction, particularly in the case of chronic KD or tibiofemoral subluxation [42, 45, 48, 54, 55, 56]. While hinged knee braces can also offer support and early controlled ROM, a chronic KD presents a more complicated situation that may instead require the use of a HEF. The chronically dislocated knee requires extensive release of scar tissue and capsule, which may in turn place excessive stress on reconstructed ligaments. HEF can potentially reduce this stress following release by maintaining the reduction while re‐establishing stable anatomic ROM, thereby protecting the reconstructed ligaments [42, 48].

The purpose of this study was to systematically review the clinical indications and considerations for HEF in the management of KDs, tibiofemoral subluxations and knee fracture‐dislocations.

## METHODS

This systematic review was conducted in accordance with the 2020 Preferred Reporting Items for Systematic Reviews and Meta‐Analyses (PRISMA) statement [41]. Two reviewers (E.A. and S.L.) conducted the systematic review independently, and a third reviewer (M.M.) was consulted to resolve any disagreements.

### Search Strategy

The protocol for the systematic review was established prior to the initiation of the search. A systematic literature search was conducted in PubMed, CINAHL (Cumulated Index to Nursing and Allied Health Literature), Cochrane, Scopus and SPORTDiscus, with a filter set to the English language and no limitations on publication date or country. The initial search was conducted on 20 October 2024 by E.A. The search was repeated on 3 November 2024 by S.L. and yielded the same results. A full list of search terms used for each database can be seen in Appendix A. Other databases, such as Embase and Web of Science, were not included due to the overlapping coverage with the other selected databases. A search of the grey literature was also performed to identify unpublished data or articles that are not catalogued or indexed by searching both ‘knee’ and ‘hinged external fixator’ on Google, Google Scholar, ClinicalTrials.gov, International Clinical Trials Registry Platform (ICTRP), Trip database and on websites for the following organizations: the American College of Surgeons, the American Surgical Association, the American Society of General Surgeons and the American Academy of Orthopaedic Surgeons.

### Study inclusion and exclusion criteria

Comparative or non‐comparative studies on patients with a HEF applied for an acute or chronic KD, tibiofemoral subluxation, or knee fracture‐dislocation were included. Non‐comparative studies, including descriptive case series and case reports, were included due to the relative novelty and rarity of HEF, as the limited number of patients treated with these devices makes it challenging to conduct high‐volume comparative studies. Exclusion criteria were the following: (1) a HEF was not used; (2) a non‐hinged external fixator or a brace/splint/immobilizer was used instead of a HEF; (3) a HEF was used on a joint other than the knee; (4) a HEF was used on the knee for an injury other than a KD (e.g., a tibial plateau fracture or an isolated ligamentous injury); and (5) the study was a review article, commentary, conference abstract, or animal or cadaveric study. E.A. and S.L. independently conducted the study selection based on the inclusion and exclusion criteria. M.M. was consulted to resolve any disagreements through additional discussion.

### Data extraction

The following items were extracted from all included studies: (1) study information, including first author name and publication year; (2) study design, including type of study and number of patients with a KD treated with a HEF; (3) patient demographics, including age and sex; (4) injury characteristics, including KD classifications using the Schenck system, injured structures, mechanism of injury, whether the injury was open, associated neurovascular injuries, associated fractures; and (5) treatment, including the type of HEF used, the timing and duration of HEF application, and explicitly described indications for the application of the HEF. The following primary (1–3) and secondary (4–8) indications were selected for analysis based on this data extraction methodology: (1) acute and chronic KD; (2) acute and chronic tibiofemoral subluxation; (3) knee fracture‐dislocation; (4) associated vascular or soft‐tissue injury; (5) associated extensor mechanism disruption; (6) status post extensive capsular release for arthrofibrosis; (7) combined osseous and ligamentous deficiency; and (8) morbid obesity. Data were collected independently by two authors (E.A. and S.L.), referencing a third author (M.M.) for further discussion on any discrepancies or disagreements.

### Risk of bias

The quality assessment of the included studies was performed using the Methodological Index for Nonrandomized Studies checklist, where each item is scored on a scale of 0–2. The maximum score for a noncomparative study is 16, while for a comparative study, the maximum score is 24. A score of 0 < *X* < 6 indicates very low quality, 6 ≤ X < 10 indicates low quality, 10 ≤ *X* < 14 indicates fair quality, and *X* > 15 indicates good quality. For randomized studies, the Jadad Scale (Oxford Quality Scoring System) was used, where the maximum score possible is 5, and a score of 3 or greater is considered good quality.

### Strength of evidence

The strength of evidence for each clinical indication for HEF was assessed using a systematic and standardized approach based on the Oxford Centre for Evidence‐Based Medicine (OCEBM) Levels of Evidence 2.1 framework [39]. This framework categorizes the quality of evidence from Level 1 (highest quality) to Level 5 (lowest quality), considering the study design, methodological rigour, and applicability to clinical practice. Each of the 14 included studies was initially assigned an evidence level based on its design. This categorization allowed for an initial stratification of evidence quality according to the OCEBM guidelines. Next, each study was assessed for its relevance to the indications for HEF and mapped to at least one indication based on the explicit rationale and clinical context described in the study. The strength of evidence for each indication was determined by evaluating the number and quality of studies supporting the indication as well as the specificity and detail of the clinical rationale provided. Studies with higher methodological rigour, such as randomized controlled trials (RCTs) and prospective cohort studies, were weighted more heavily than case series and case reports.

Evidence for each indication was categorized as strong, moderate or weak using a best‐evidence synthesis approach adapted from the methodology presented in another systematic review [10]. Indications were considered to have strong evidence if supported by more than one Level 1 or 2 study. Moderate evidence was assigned to an indication when supported by only one Level 1 or 2 study. Weak evidence was assigned when the indication was not supported by any Level 1 or 2 studies.

## RESULTS

### Search results

**Figure 2:**
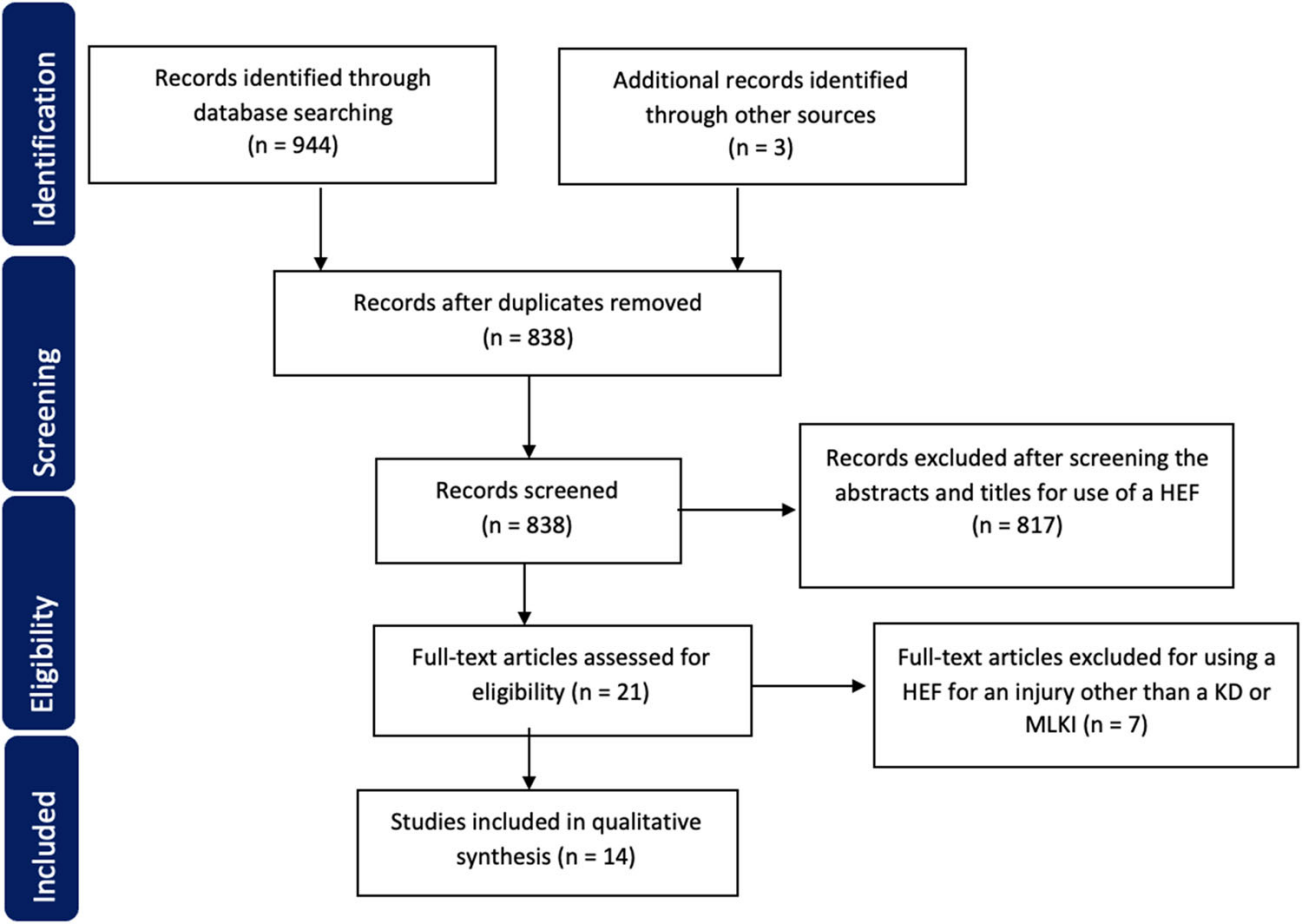
PRISMA flow diagram of the literature search. HEF, hinged external fixation; KD, knee dislocation; MLKI, multi‐ligamentous knee injury; PRISMA, Preferred Reporting Items for Systematic Reviews and Meta‐Analyses.

A search query in PubMed, CINAHL, Cochrane, Scopus and SPORTDiscus yielded a pooled result of 944 articles, with three additional articles added manually after a search of the Grey Literature. As shown in Figure 2, after 109 duplicate articles were removed, 838 articles were screened, and 817 articles were excluded based on the study title and abstract. Of the 21 remaining, 14 studies were eligible for inclusion in the systematic review and further analysis after full‐text review (Figure 2).

The 14 included studies consisted of a mix of case reports, case series, a prospective cohort study, and RCTs, with a total of 184 knees treated with a HEF for a KD, tibiofemoral subluxation or knee fracture‐dislocation. Pertaining to the five case reports included, the indications described for the application of HEF included: (1) two cases of chronic fixed posterior KDs with status post extensive capsular release for arthrofibrosis; (2) a chronic posterior KD and status post extensive capsular release for arthrofibrosis 8 weeks after an initial fracture‐dislocation (initially treated with tibial nailing and a plaster cast and then 4 weeks later with anterior cruciate ligament (ACL) and posterior cruciate ligament (PCL) reconstruction and application of a hinged knee brace); (3) following bi‐cruciate ligament reconstruction after an acute KD to aid in early ROM to prevent arthrofibrosis; (4) a case of a chronic coronal tibiofemoral subluxation; and (5) following ligament reconstruction after a fracture‐dislocation with extensor mechanism injury to help aid in early ROM. The RCTs and cohort study included patients with HEF application for acute or chronic KDs and fracture‐dislocations. The case series all included patients with HEF for acute or chronic KDs, with one study specifying the use of HEF for vascular injury and extensor mechanism injury, and another study specifying the use of HEF for chronic subluxation, fracture dislocation, morbid obesity and status post extensive capsular release for arthrofibrosis. Characteristics of the included studies can be found below in Table 1, and characteristics of the included injuries are found in Table 2.

**Table 1:** Characteristics of studies on hinged external fixation about the knee.

Author (year)	Study design	Level of evidence	Device(s) used	Relevant indications^a^	MINORS score	Jadad score
Simonian et al. (1998) [48]	Case report	V	Compass Elbow Hinge [50]	1, 6	9/16	N/A
Richter and Lobenhoffer (1998) [45]	Case report	V	Compass Elbow Hinge	1, 3, 6	8/16	N/A
Stannard et al. (2003) [55]	Prospective cohort study	II	Compass Knee Hinge [38]	1, 3	22/24	N/A
Zaffagnini et al. (2008) [58]	Case report	V	Citieffe ST.A.R.90 F4 [8]	1	6/16	N/A
Marcacci et al. (2012) [39]	Case series	IV	Citieffe ST.A.R.90 F4	1	13/24	N/A
Stannard et al. (2014) [54]	RCT	I	Compass Knee Hinge	1	N/A	5/5
Angelini et al. (2015) [3]	Case series	IV	Orthofix Knee Hinge (LRS Advanced System) [40]	1	13/16	N/A
Angelini et al. (2015) [2]	RCT	I	Orthofix Knee Hinge (LRS Advanced System)	1	N/A	5/5
Stannard (2016) [53]	Case series	IV	Compass Knee Hinge	3	13/16	N/A
Geiger et al. (2016) [18]	Case report	V	Compass Universal Hinge	2	12/16	N/A
Liu et al. (2017) [27]	Case series	IV	Unspecified	1, 4, 5	10/16	N/A
Takahashi et al. (2019) [56]	Case report	V	Hinged Ilizarov Frame	1, 3, 5	8/16	N/A
Sobrado et al. (2022) [51]	Case series	IV	Unspecified	1	17/24	N/A
Pardiwala et al. (2023) [42]	Case series	IV	Hinged Ilizarov Frame and Compass Universal Hinge	1, 2, 3, 6, 8	10/16	N/A

Abbreviations: MINORS, Methodological Index for Nonrandomized Studies; RCT, randomized controlled trial.

^a^ 1: Acute and Chronic Knee Dislocation, 2: Acute and Chronic Tibiofemoral Subluxation, 3: Knee Fracture‐Dislocation, 4: Associated Vascular or Soft Tissue Injury, 5: Associated Extensor Mechanism Disruption, 6: Status Post Extensive Capsular Release for Arthrofibrosis, 7: Combined Osseous and Ligamentous Deficiency, 8: Morbid Obesity.

**Table 2:** Patient characteristics of studies on hinged external fixation about the knee.

Author (year)	# of Knees	Age (years)	Sex (M/F)	KD classification (#)	Open injuries (#)	Mechanism of injury (#)	Neurovascular injuries (#)	Associated fractures (#)	Time from injury to application in weeks (range)	Duration of device application in weeks	Follow‐up duration in months (range)
Simonian et al. (1998) [48]	2	17	0/2	KD‐III‐L (2)	0	Low‐Energy (1), Ultra Low‐Energy (1)	Peroneal nerve (1)	0	16, 32	6	12, 15
Richter and Lobenhoffer (1998) [45]	1	17	0/1	KD‐II (1)	1	High‐Energy (1)	0	Tibia (1)	8	6	12
Stannard et al. (2003) [55]	12	32^a^	26/14^a^	NA	1^a^	High‐Energy (36)^a^	Peroneal nerve (4) Popliteal artery (4)^a^	Tibial plateau (unspecified)^a^	4 (1–21)	6–8	24 (14–41)^a^
Zaffagnini et al. (2008) [58]	1	47	1/0	KD‐IV (1)	0	High‐Energy (1)	0	NA	1	4	NA
Marcacci et al. (2012) [39]	8	29	7/1	KD‐IV (4), KD‐V.4 (2), KD‐V.2 (1), KD‐V.3M (1)	3	High‐Energy (8)	Popliteal artery (1)	Tibial plateau (3) Patella (1)	NA	4	26 (10–45)
Stannard et al. (2014) [54]	47	35.6	34/12	KD‐I (2), KD‐III (25), KD‐IV (20)	5	High‐Energy (41), Low‐Energy (6)	Popliteal artery (7), Peroneal nerve (unspecified)^a^	Acetabulum (unspecified), Tibial plateau (unspecified)^a^	NA	6	39 (12–86)^a^
Angelini et al. (2015) [3]	14	29	NA	KD‐III‐L (9), KD‐III‐M (3), KD‐IV (2)	NA	High‐Energy (11), Low‐Energy (3)	0 (exclusion criterion)	0 (exclusion criterion)	11 (2–12)	6	49 (41–58)
Angelini et al. (2015) [2]	15	29	12/3	KD‐III‐L (14), KD‐III‐M (1)	NA	NA	NA	0	Sub‐Acute (>3 weeks) or Chronic (>3 months)	6	27 (14–51)
Stannard (2016) [53]	30	NA	NA	KD‐V (30)	NA	NA	NA	Tibial plateau (30)	(3–4)	6‐8	42 (25–58)
Geiger et al. (2016) [18]	1	51	0/1	N/A	0	High‐Energy (1)	0	Pelvic fracture, Contralateral femoral shaft fracture, Cavitary impaction fracture of the tibial eminence	6	6	60
Liu et al. (2017) [27]	15	29	13/2	KD‐I‐AM (1), KD‐III‐M (3), KD‐III‐L (1), KD‐IV (3), KD‐V.2 (1), KD‐V.3M (1), KD‐V.3L (1)	NA	High‐Energy (14), Low‐Energy (1)	Popliteal artery (4), Peroneal nerve (2), Tibial nerve (2)	Patella (11)	NA (19 days after vessel repair)	6+	36 (14–60)
Takahashi et al. (2019) [56]	1	18	1/0	KD‐V.4 (1)	1	High‐Energy (1)	Peroneal nerve (1)	Patella (1)	0	7	24
Sobrado et al. (2022) [51]	16	39^a^	52/10^a^	KD‐III‐L (43), KD‐III‐M (8), KD‐IV (11)^a^	NA	NA	Popliteal artery (3), Peroneal nerve (9)^a^	0 (exclusion)^a^	31^a^	6^a^	72^a^
Pardiwala et al. (2023) [42]	21	29	17/4	NA	NA	High‐Energy (19), Low‐energy (2)	Popliteal artery (4), Peroneal nerve (5)	NA	11 (4–22)	NA	83 (32–194)

*Note*: In most instances where NA is used, the original articles did not report on the associated variables, such as certain follow‐up data and specific KD classifications. Some studies did not collect these details at all, while others omitted relevant information in the text, such as the exact number of knees falling under a specific classification. As a result, these fields could not be reliably extracted and are thus indicated as NA.

Abbreviations: KD, knee dislocation; N/A, not applicable; NA, not available.

^a^ The study does not specify whether the mean/count for this variable was for the brace or hinged external fixator group. Thus, these reported values include not just patients treated with hinged external fixation but also those treated with a brace.

Among the primary and secondary clinical indications assessed, acute and chronic KD and associated vascular or soft‐tissue injury were supported by the strongest evidence, with three and two studies of higher methodological quality contributing to these findings, respectively (Table 3). Explicitly discussed clinical rationales for HEF application in these higher quality studies included: (1) to protect articular and/or ligamentous repairs at least as efficiently as rigid immobilization while simultaneously allowing ROM exercises to be initiated immediately; (2) to minimize the increased risk of knee pain, stiffness, and dysfunction in patients with more than two ligaments injured and those who received acute surgery [2, 54, 55]. Indications, including knee fracture‐dislocation and combined osseous and ligamentous deficiency, were assigned moderate evidence, each with one study of higher methodological quality contributing to the indication. The remaining indications were assigned weak evidence due to the absence of studies of higher methodological quality, with findings derived only from case reports and/or series.

**Table 3:** Clinical indications for hinged external fixation about the knee.

Indication		Number of knees	Relevant studies	Strength of evidence
Primary	Acute and chronic knee dislocation	138	Stannard et al. [54], Stannard et al. [55], Zaffagnini et al. [58], Marcacci et al. [30], Richter and Lobenhoffer [45], Angelini et al. [3], Takahashi et al. [56], Simonian et al. [48], Angelini et al. [2], Sobrado et al. [51], Pardiwala et al. [42], Liu et al. [27]	Strong
	Acute and chronic tibiofemoral subluxation	2	Geiger et al. [18], Richter and Lobenhoffer [45]	Weak
	Knee fracture‐dislocation	39	Marcacci et al. [30], Stannard [53], Liu et al. [27]	Moderate
Secondary	Associated vascular or soft‐tissue injury	38^a^	Stannard et al. [55], Stannard et al. [54], Marcacci et al. [30], Richter and Lobenhoffer [45], Simonian et al. [48], Takahashi et al. [56], Sobrado et al. [51], Pardiwala et al. [42], Liu et al. [27]	Strong
	Associated extensor mechanism disruption	16	Takahashi et al. [56], Liu et al. [27]	Weak
	Status post‐extensive capsular release for arthrofibrosis	23	Simonian et al. [48], Pardiwala et al. [42]	Weak
	Combined osseous and ligamentous deficiency	40	Marcacci et al. [30], Richter and Lobenhoffer [45], Stannard [53], Liu et al. [27]	Moderate
	Morbid obesity	2	Pardiwala et al. [42]	Weak

^a^ Sobrado et al. did not specify whether the neurovascular injuries were in the brace or hinged external fixator group. Thus, all reported neurovascular injuries were included in this number.

## DISCUSSION

The main finding of this review is that HEF, though far less common than rigid knee‐spanning external fixation (KSEF), has been utilized in the management of KDs that involve combined osseous and ligamentous deficiency, extensive capsular release for arthrofibrosis, vascular or soft‐tissue compromise, and/or extensor mechanism disruption. HEF appears to be a potentially valuable tool for the orthopaedic surgeon in treating these injuries to enhance joint stability while enabling progressive rehabilitation, particularly in cases where early controlled ROM is needed to minimize complications like arthrofibrosis and recurrent instability. However, there remains no clear consensus on the indications or standardized protocols for HEF application, largely due to the limited level of evidence, small sample sizes, and absence of comparative studies. As such, its routine or widespread use cannot yet be firmly recommended but should be considered by surgeons managing these complex knee soft tissue and osseous injuries.

**Figure 3:**
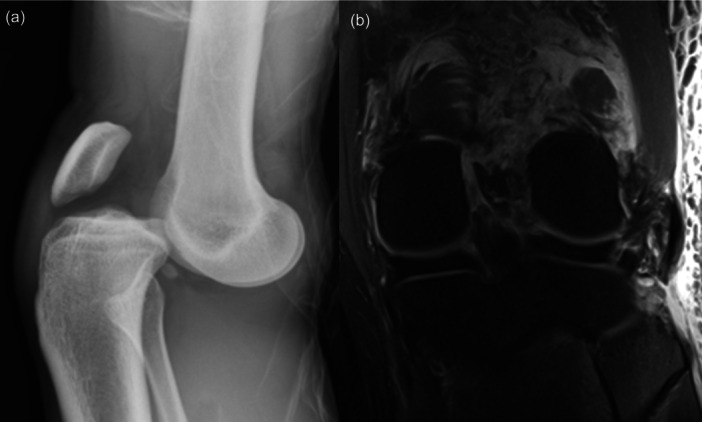
(a) X‐ray revealing an anterior knee dislocation. (B) Coronal T2‐weighted MRI image showing a KD‐III‐L with avulsion of the biceps femoris and lateral collateral ligament from the fibular head. KD, Knee dislocation; MRI, magnetic resonance imaging.

**Figure 4:**
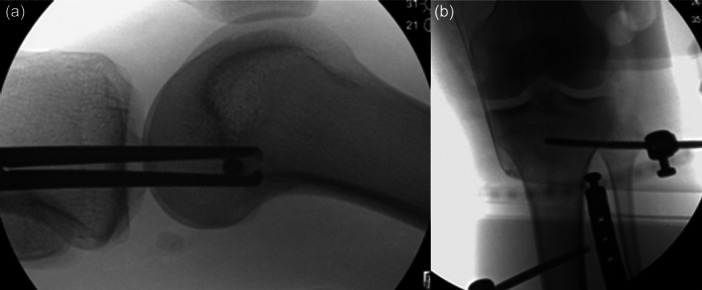
(a) Lateral fluoroscopic image demonstrating the placement of the central axis reference pin off of which the hinged external fixator will be based. (B) AP radiograph demonstrating the hinged external fixator in place after the central axis reference pin has been removed. AP, anterior–posterior.

Overall, there is a lack of consensus in the literature as to the optimal treatment approach for KDs, and several controversial post‐operative immobilization strategies exist [2, 3, 15, 26, 28, 32, 57]. KDs and MLKIs are often burdened with high rates of knee stiffness, with 12.1% of patients diagnosed with post‐operative arthrofibrosis [12]. While the severity of injury itself is likely a predisposing factor for arthrofibrosis, the mechanism of immobilization must be considered [32]. Non‐invasive immobilization includes bracing or casting, while invasive methods include KSEF, which typically takes the form of rigid external fixation and therefore carries the added burdens of cost, utilization of hospital resources and patient morbidity. Extended immobilization in a rigid external fixation device may also predispose the knee to arthrofibrosis, which can have devastating long‐term consequences for the patient, such as pain and functional impairment due to stiffness and restricted ROM [12, 25]. Therefore, the risks of rigid external fixation need to be weighed against the benefits of greater stability, visibility and accessibility of the surrounding soft‐tissue envelope [32]. HEF as an alternative to rigid external fixation offers the potential capability of early controlled mobilization of the knee as well as multiplanar joint stability and soft‐tissue accessibility [32]. Several studies describe the use of HEF in the setting of a KD [2, 3, 27, 30, 42, 45, 48, 51, 54, 55, 56, 58]. Despite these potential advantages, its use remains limited. When managing the spectrum of KDs and MLKIs, the surgeon needs to be knowledgeable and capable of utilizing this modern technology. An example of an acute anterior KD where HEF was utilized to support the acute posterolateral corner (PLC) repair is seen below in Figure 3, followed by an example of accurate positioning of the central axis of rotation in Figure 4.

**Figure 5:**
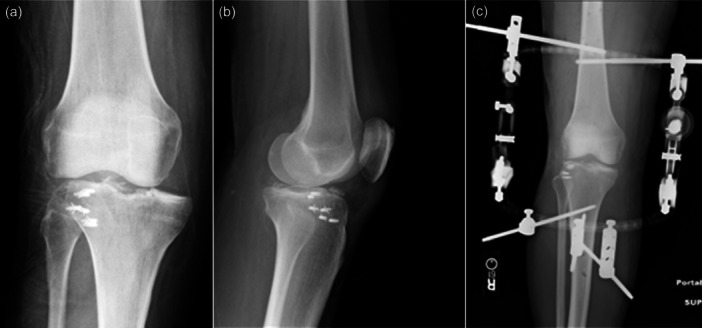
(a) Anteroposterior and (b) lateral radiographs at initial presentation show substantial subacute medial tibial subluxation in coronal plane but not sagittal plane. (C) Post‐operative radiograph shows application of hinged knee external fixator after tibiofemoral reduction.

**Figure 6:**
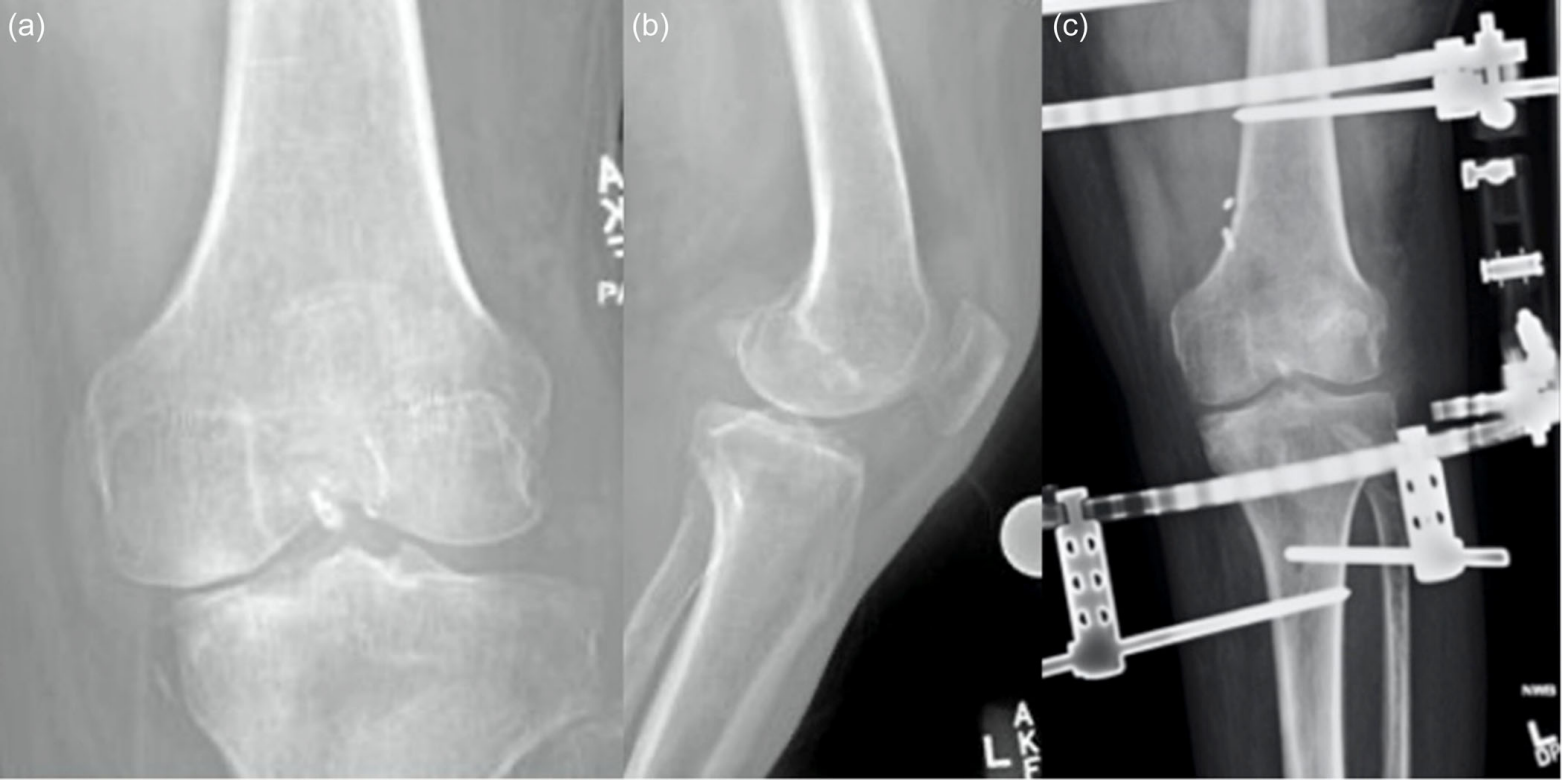
(a) Anteroposterior and (b) lateral radiographs at initial presentation 9 months post‐injury showing substantial lateral tibial subluxation in the coronal plane only. (C) Post‐operative radiograph shows application of hinged external fixator after tibiofemoral reduction and anterior cruciate ligament/posterolateral corner reconstruction.

As for acute or chronic tibiofemoral subluxations, Maak et al. describe an operative management plan that involves extensive release of scar tissue to achieve anatomic reduction, followed by reconstruction of cruciate ligaments and other injured intraarticular structures, with subsequent application of a hinged knee brace or HEF [28]. Geiger et al. describe the use of a HEF for a patient who developed coronal tibiofemoral subluxation following MLKI and fracture of the tibial eminence (Figure 5) [18]. The patient had been initially treated with an en masse PLC repair and then placed into a hinged knee brace. Follow‐up care closer to home at six weeks post‐injury demonstrated medial tibiofemoral subluxation. While the medial collateral ligament (MCL) was intact, HEF was indicated due to progressive medial subluxation and resultant instability despite the initial use of rigid external fixation. HEF functioned to maintain reduction and coronal alignment of the tibiofemoral joint, thereby protecting the repaired PLC and native MCL while allowing controlled ROM. HEF may therefore be indicated in the settings of both acute and chronic (Figure 6) tibiofemoral subluxation.

**Figure 7:**
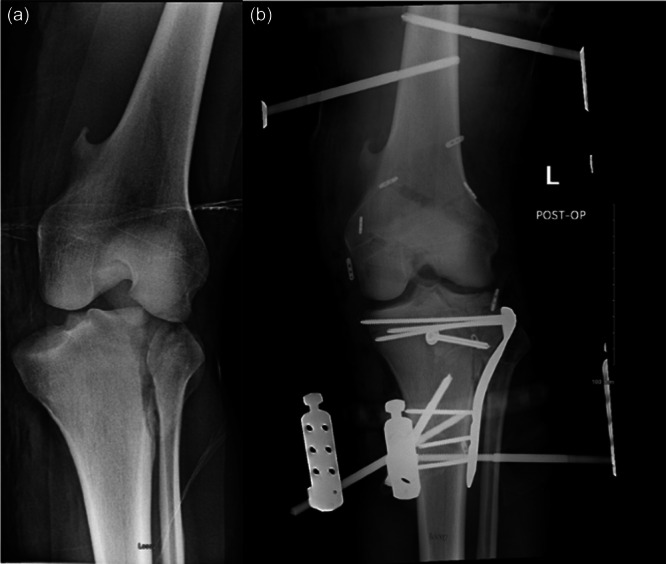
(a) AP radiograph demonstrating lateral tibial plateau split fracture with dislocation of the medial plateau. (B) AP radiograph demonstrating the reduced and stabilized lateral tibial plateau, multiligamentous reconstruction and application of hinged external fixation. AP, anterior–posterior.

Finally, for knee fracture‐dislocations, Stannard et al. describe a staged surgical treatment algorithm using a HEF [9, 36, 53]. The first step involves fracture reduction and fixation, ideally within one week of the initial injury depending on the condition of the soft‐tissue envelope. KSEF, rather than a knee immobilizer, may be indicated in this initial stage if there is continued tibiofemoral instability of either subluxation or dislocation. Next, associated ligaments are reconstructed in a staged fashion, 3–4 weeks after the initial injury, with the PCL and posteromedial corner (PMC) reconstructed during the initial surgery. A HEF device is applied at the time of the ligament reconstructive surgery to enhance post‐operative stability and mobility. The surgeon managing these injuries needs to make a surgical determination of whether any additional invasive external immobilization is needed and, if so, whether there is a need for additional multiplanar support for potential coronal and/or sagittal plane instability. The difficulty of establishing an algorithm for the treatment of knee fracture‐dislocations lies in the heterogeneous nature of both the fracture patterns and ligament injuries. Periarticular fractures can vary in size, position and their relative contribution to tibiofemoral instability. Similarly, ligament injury location may play a role in the treatment options of repair or reconstruction, and the relative stabilizing effect of ligament versus osseous treatment may vary significantly between injury patterns. The role of HEF in these combined bony and soft tissue injuries is not yet determined, but may be needed to support an unstable osseous fracture pattern and osteosynthesis while also attempting to protect or unload a ligamentous repair or reconstruction during the early healing of these stabilizing structures. HEF may therefore be an integral component of staged treatment algorithms for complex knee injuries. An example of a HEF being used for a knee fracture‐dislocation along with internal fixation of the tibial plateau fracture can be seen in Figure 7.

There are several potential complications associated with HEF of the knee. Pin site issues are common, including infection, fracture and pain [11, 24, 52, 59]. Device‐related biomechanical problems can also occur, such as pin breakage, loosening or malalignment of the fixator, which can affect joint kinematics and rehabilitation progress [16]. Technical challenges exist with accurate application, particularly with locating the true axis of rotation of the knee joint for proper hinge alignment [16, 23, 30, 48, 52, 55]. Malalignment can lead to abnormal joint stresses from inadvertent compression, distraction, or subluxation, which can impair ligament or osteosynthesis healing or reconstruction outcomes [16]. The technically demanding nature of HEF application may limit its widespread use. For further in‐depth description of the technique of placing a hinged knee external fixator, the following is recommended as a definitive reference [38]. Additionally, the bulky external hardware may cause aesthetic and psychological distress for patients, which may impact treatment adherence and quality of life during the recovery period [1, 21]. It has been demonstrated that patients with strong social support can better manage the stressors of external fixation [44]. In the absence of absolute indications, such as those described in this review, the impact of HEF on patients' psychological well‐being should be considered a key factor in the treatment plan [21]. HEFs require close clinical follow‐up and monitoring, with frequent adjustments often needed to maintain proper alignment and function. Such intensive follow‐up can be burdensome for patients and healthcare providers. These complications highlight the need to carefully consider HEF indications and alternatives, as the potential benefits of early mobilization and protected ROM must be weighed against the risks of complications and the impact on patient comfort and adherence.

Overall, the present findings suggest that HEF may be highly beneficial in specific scenarios where prolonged rigid immobilization could compromise long‐term function by increasing the risk of stiffness and arthrofibrosis. This includes patients requiring staged ligament reconstruction and/or extensive capsular release for arthrofibrosis. However, careful patient selection and precise application are essential to avoid complications such as improper hinge alignment, pin‐site infection or significant psychological distress. While HEF is not yet a routine tool in the management of complex knee injuries, its ability to protect ligament repairs and reconstructions after definitive treatment while enabling controlled ROM is well‐established by multiple high‐quality studies [2, 54, 55]. Further research should focus on high‐quality prospective studies assessing outcomes of HEF compared to other forms of immobilization. Comparing functional and biomechanical outcomes between hinged and rigid external fixation following these injuries is needed to assess the theoretical added benefit of HEF for joint stability and mobility and prevention of arthrofibrosis. Additionally, long‐term outcomes of HEF across various indications should be investigated to clarify the optimal timing and duration of HEF application, as well as enable the assessment of HEF in specific patient populations, such as those with morbid obesity or neglected chronic KDs. Future work should also take care to standardize the reporting of injury characteristics to facilitate evidence synthesis.

There are several limitations to this systematic review. First, the included studies are heterogeneous in design, including five Level V and six Level IV articles. Thus, most of the included studies have small sample sizes and/or no comparison group. Second, several included studies had no clear mention of the specific rationale for HEF application, requiring deduction of the indications through assessment of patient characteristics. Third, the sums of each indication provided in Table 3 are broadly underestimated due to underreporting of HEF indications and injury characteristics. Without detailed neurovascular injury characteristics, it is difficult to assess whether HEF was primarily used as a temporizing measure for vascular injuries or as a definitive solution for ligament reconstruction. Fourth, there was inconsistent use of the Schenck KD classification system across studies, with some using the PLC and PMC, and others using the lateral collateral ligament and MCL, which limits the reliability of comparisons across studies. Fifth, three of the included studies compared patients treated with HEF to those treated with a brace but did not provide statistics separately for the HEF and brace groups. Thus, some of the variables found in Table 2 for these studies include means and counts for all patients in the study rather than just those treated with a HEF.

## CONCLUSION

This systematic review evaluated the clinical indications and considerations for HEF in the treatment of KDs, tibiofemoral subluxations and knee fracture‐dislocations. There is currently no consensus on the specific indications for HEF in the setting of complex knee injuries, despite the significant risk of recurrent instability, stiffness and arthrofibrosis. While additional prospective studies are needed, the current literature suggests that HEF appears to be a valuable option in the treatment armamentarium of the surgeon involved in the management of KDs with combined osseous and ligamentous deficiency or significant soft‐tissue and vascular injury.

## AUTHOR CONTRIBUTIONS

**Ekrem M. Ayhan**: Writing—review and editing; writing—original draft; methodology; formal analysis; data curation; conceptualization. **Sarah Levitt**: Writing—review and editing; methodology; formal analysis; data curation; conceptualization. **Geoffrey D. Abrams**: Writing—review and editing. **James P. Stannard**: Writing—review and editing. **Michael J. Medvecky**: Writing—review and editing; methodology; formal analysis; data curation; conceptualization; supervision.

## CONFLICT OF INTEREST STATEMENT

Michael J. Medvecky serves as a speaker for educational symposia sponsored by DePuy Mitek and Smith & Nephew. Geoffrey D. Abrams serves as a consultant for Bioventus and Health and Human Services; receives payment for expert testimony from Health and Human Services and Private Law Offices; has a Collaged Scaffold Patent; Participates on Data and Safety Monitoring Board or Advisory Board for Cytonic, Sparta Biosciences, Axgen, Arthrotak and Arthrex; serves as a committee member for AAOS, AOSSM and ASES; has stock ownership in Cytonics, Axgen and Arthrotak; receives equipment/materials/gifts/other services from Arthrex. James P. Stannard receives grants/contracts from Arthrex, NIH (NIAMS & NICHD), and the US Department of Defence; royalties/licences from Thieme; consulting fees from Arthrex, DePuy, Orthopaedic Designs North America and Smith & Nephew; serves in a leadership role at the Journal of Knee Surgery. The remaining authors declare no conflicts of interest.

## ETHICS STATEMENT

This investigation was deemed exempt under Yale University IRB 2000028912.

## Data Availability

All data generated and/or analyzed during this study are included in this published article and its Appendix.

## Appendix

See Table A1.

**Table A1:** Search strategies.

Database	Search terms	Search result
PubMed	((("External Fixators"[Mesh]) AND (hinge)) OR ((hinged external fixator) OR (hinged external fixation) OR (compass knee hinge) OR (articulated external fixator) OR (dynamic external fixator))) AND (("Knee Joint"[Mesh] OR "Knee"[Mesh] OR "Knee Dislocation"[Mesh] OR "Knee Injuries"[Mesh] OR "Knee Fractures"[Mesh]) OR (Knee))	803
CINAHL	(knee* OR ((MH "Knee") OR (MH "Knee Injuries+") OR (MH "Knee Fractures+") OR (MH "Knee Dislocation+") OR (MH "Knee Surgery+") OR (MH "Knee Joint+") OR (MH "Knee Injuries, Articular Cartilage"))) AND (((hinged external fixator*) OR (hinged external fixation*) OR (compass knee hinge*) OR (articulated external fixator*) OR (dynamic external fixator*)) OR (hinge* AND ((MH "External Fixators+"))))	20
Scopus	(TITLE‐ABS‐KEY(Knee*) AND TITLE‐ABS‐KEY((hinged external fixator*) OR (hinged external fixation*) OR (compass knee hinge*) OR (articulated external fixator*) OR (dynamic external fixator*)))	97
Cochrane	(#1) (knee*):ti,ab,kw (44214)	11
	(#2) MeSH descriptor: [Knee Dislocation] explode all trees (12)	
	(#3) MeSH descriptor: [Knee] explode all trees (1118)	
	(#4) MeSH descriptor: [Knee Injuries] explode all trees (1969)	
	(#5) MeSH descriptor: [Knee Joint] explode all trees (5042)	
	(#6) MeSH descriptor: [Knee Fractures] explode all trees (23)	
	(#7) #1 OR #2 OR #3 OR #4 OR #5 OR #6 (44462)	
	(#8) ((hinged external fixator*) OR (hinged external fixation*) OR (compass knee hinge*) OR (articulated external fixator*) OR (dynamic external fixator*)):ti,ab,kw (40)	
	(#9) (hinge*):ti,ab,kw (426)	
	(#10) MeSH descriptor: [External Fixators] explode all trees (1366)	
	(#11) #9 AND #107	
	(#12) #8 OR #11 (45)	
	(#13) #7 AND #1211	
SPORTDiscus	(((hinged external fixator*) OR (hinged external fixation*) OR (compass knee hinge*) OR (articulated external fixator*) OR (dynamic external fixator*)) OR ((DE "TREATMENT of fractures") AND hinge*)) AND ((DE "KNEE" OR DE "ANTERIOR cruciate ligament" OR DE "KNEE joint" OR DE "MEDIAL collateral ligament (Knee)" OR DE "PATELLA" OR DE "POSTEROLATERAL corner" OR DE "KNEE braces" OR DE "KNEE diseases" OR DE "ILIOTIBIAL band syndrome" OR DE "PATELLA diseases" OR DE "KNEE exercises" OR DE "KNEE injuries" OR DE "ANTERIOR cruciate ligament injuries" OR DE "PATELLAR ligament injuries" OR DE "PLICA syndrome" OR DE "POSTERIOR cruciate ligament injuries" OR DE "KNEE injury treatment" OR DE "KNEE joint" OR DE "MENISCUS (Anatomy)" OR DE "PATELLOFEMORAL joint" OR DE "KNEE pain" OR DE "KNEE physiology" OR DE "KNEE surgery" OR DE "ANTERIOR cruciate ligament surgery" OR DE "AUTOLOGOUS chondrocyte implantation" OR DE "MICROFRACTURE surgery" OR DE "PATELLAR ligament surgery" OR DE "POSTERIOR cruciate ligament surgery" OR DE "TOTAL knee replacement") OR knee*)	13
Manual addition via Grey Literature search		3
Total		947
